# Local dynamic stability of the lower-limb as a means of post-hoc injury classification

**DOI:** 10.1371/journal.pone.0252839

**Published:** 2021-06-04

**Authors:** Jacob Larson, Edmon Perkins, Taylor Oldfather, Michael Zabala

**Affiliations:** 1 Department of Mechanical Engineering, Auburn University, Auburn, Alabama, United States of America; 2 Department of Mechanical & Aerospace Engineering, North Carolina State University, Raleigh, North Carolina, United States of America; Tokai University, JAPAN

## Abstract

Since most sporting injuries occur at the lower extremity (50% to 66%) and many of those injuries occur at the knee (30% to 45%), it is important to have robust metrics to measure risk of knee injury. Dynamic measures of knee stability are not commonly used in existing metrics but could provide important context to knee health and improve injury screening effectiveness. This study used the Local Dynamic Stability (LDS) of knee kinematics during a repetitive vertical jump to perform a post-hoc previous injury classification of participants. This study analyzed the kinematics from twenty-seven female collegiate division 1 (D1) soccer, D1 basketball, and club soccer athletes from Auburn University (height = 171 ± 8.9cm, weight = 66.3 ± 8.6kg, age = 19.8 ± 1.9yr), with 7 subjects having sustained previous knee injury requiring surgery and 20 subjects with no history of injury. This study showed that LDS correctly identified 84% of previously injured and uninjured subjects using a multivariate logistic regression during a fatigue jump task. Findings showed no statistical difference in kinematic position at maximum knee flexion during all jumps between previously injured and uninjured subjects. Additionally, kinematic positioning at maximum knee flexion was not indicative of LDS values, which would indicate that future studies should look specifically at LDS with respect to injury prevention as it cannot be effectively inferred from kinematics. These points suggest that the LDS preserves information about subtle changes in movement patterns that traditional screening methods do not, and this information could allow for more effective injury screening tests in the future.

## Introduction

Knee injuries come with high financial costs and decreased quality of life as well as an increased risk of developing chronic osteoarthritis [1–3]. Of an estimated 8.6 million annual sports injuries, many occur at the lower extremity (50% to 66%), and a great deal of those injuries occur at the knee (30% to 45%) [4]. Due to the high incidence of knee injury and long-lasting physical and financial consequences, it is important to have robust metrics to measure the risk of knee injury to allow for preventative measures to be taken before injuries occur.

One of the shared aspects of the metrics commonly used for evaluation of injury risk is that most techniques focus on variables that are only represented at *single points* in time (e.g., peak knee flexion, maximum knee abduction, knee valgus angle at initial contact, etc.). Although these metrics can provide valuable insights into the characteristics of an individual’s movement, they often fail to provide enough information to understand how a subject moves outside of the point of evaluation. For example, if two subjects both reach 90° of knee flexion during a jump landing, evaluating maximum knee flexion for both subjects will show them to have identical landing mechanics. However, evaluating the entire kinematic trajectory before and after maximum knee flexion could reveal that both subjects have radically different knee flexion movement patterns, which could allow for a more holistic injury risk evaluation.

One effective method of comprehensively evaluating nonlinear movement patterns is the Maximum Lyapunov Exponent (MLE), where the MLE quantifies the rate of divergence of a cyclic kinematic trajectory such as knee flexion or ankle rotation [5]. Because of the somewhat abstract nature of the MLE as a variable, it is important to contextualize the MLE depending on the task to which the MLE is being applied. Because of the design and purpose of this study, the authors will be interpreting the MLE as Local Dynamic Stability (LDS), which is consistent with other literature in this field [6–9]. For this study, the LDS is being used to quantify the sensitivity of an individual to small, intrinsic perturbations during a movement [10]. These cyclic changes can be attributed to neuromuscular noise, slight changes in terrain, or non-uniform kinematics from cycle to cycle. An increased LDS is typically associated with lower risk of injury due to the increased ability of a system to successfully adapt to perturbations or changes due to non-uniformity of motion [11]. This can especially be seen in studies that focus on elderly risk of falls, where it has been demonstrated that subjects at risk for falling exhibited lower trunk LDS than subjects not at risk for falling [12, 13].

Several biomechanical studies have identified potential lower extremity injury risk factors such as landing mechanics [14–19], gender [20–23], neuromuscular strategies [24], and anatomical features [25–27]. However, some researchers have challenged the effectiveness of these screening tests when expanded to large scale populations [28]. Because of the resources required to validate screening tests on a large scale, it can be beneficial to identify injury risk metrics using post-hoc methods of analysis between groups with previously known differences. In this way, researchers can effectively investigate new metrics for injury screening with fewer resources. Although LDS has gained popularity as a method of biomechanical analysis [10], no studies to the authors’ knowledge directly focus on the LDS as it relates to injury screening—making it a good candidate for potential validation using a post-hoc classification of previous injury. This investigation will analyze a set of athletes via post-hoc classifications based on previous knee injury history to validate the potential for the LDS to be used in future studies. Additionally, few studies have attempted to analyze the LDS of non-gait movements such as jogging or walking. While gait is more easily repeatable and simple to capture, the authors wanted to explore more dynamic movements involving jumping and landing to see if significant differences in LDS could be found. The methods and results described in this paper, although retrospective and not predictive in nature, advance knowledge needed for exploration of new predictive injury screening methods in the future.

The objective of this study was to determine the ability of LDS variables to categorize previously injured and uninjured subjects in a post-hoc classification. The authors hypothesized that lower limb LDS will be a more effective classification metric than standard kinematic variables of the lower limb, measured at maximum knee flexion.

## Methods

### Participants

Study participants included 27 female collegiate division 1 (D1) soccer, D1 basketball, and club soccer players from Auburn University (height = 171.2 ± 8.9cm, weight = 66.3 ± 8.6kg, age = 19.8 ± 1.9yr). Subjects were chosen based on anterior cruciate ligament (ACL) risk assessment, as women in cutting sports, such as soccer and basketball, have been shown to be at the greatest statistical risk for ACL injury [29]. Participants included those who were currently active players who were cleared to participate in their respective sport. Out of 27 recruited athletes, two had bilateral ACL reconstructions, two had unilateral ACL reconstructions, and three had surgical interventions for meniscus or cartilage repair. Each subject signed informed consent forms approved by the Auburn University Institutional Review Board.

### Instrumentation

**Fig 1:**
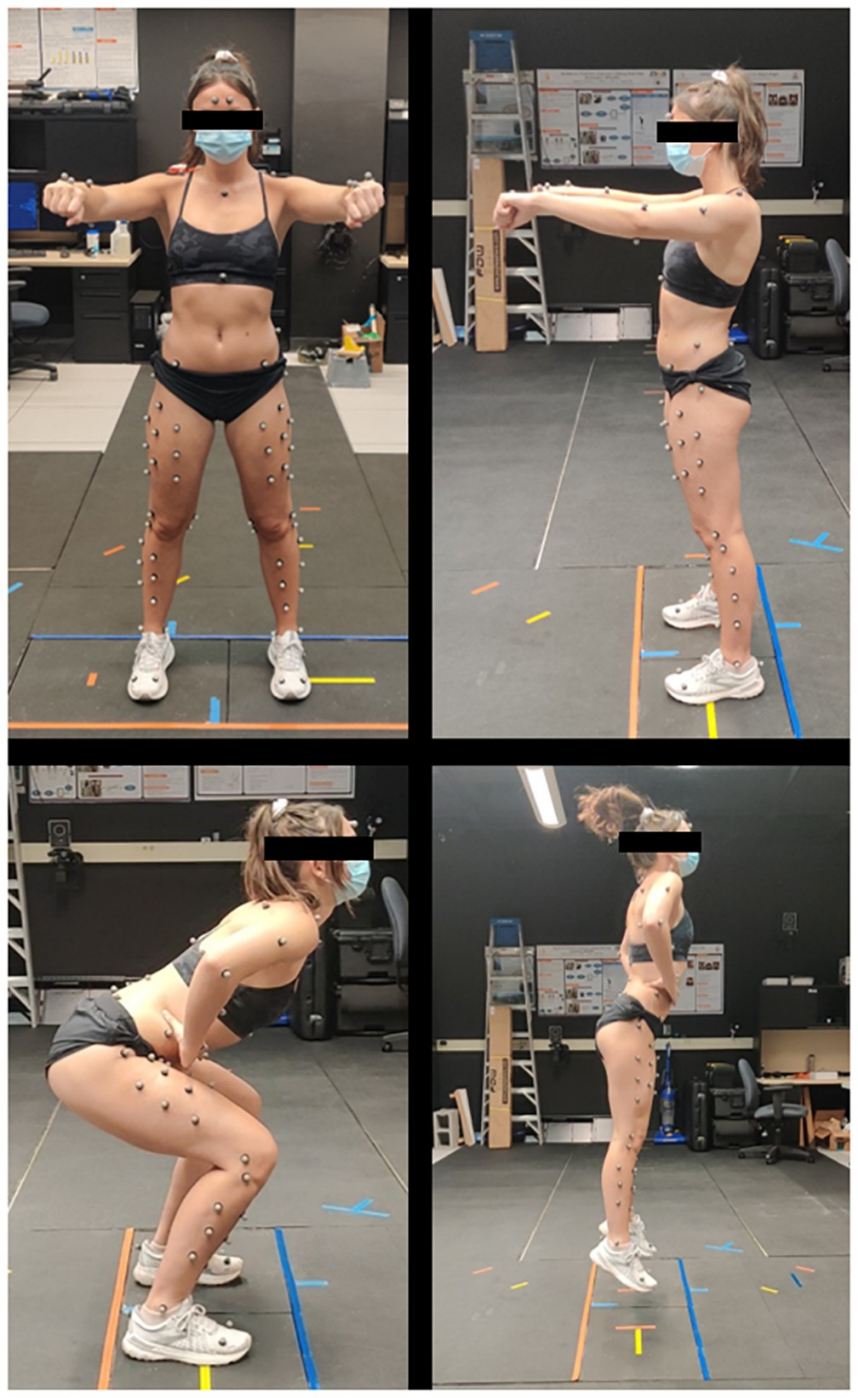
Subject marker set and task visualization. This figure shows a subject during calibration and during phases of the repeated vertical jump. Top-Left) Frontal calibration view. Top-Right) Side calibration view. Bottom-Left) Subject at maximum knee flexion during a vertical jump. Bottom-Right) Subject at peak of vertical jump.

The subjects were fitted with 79 reflective markers using the point cluster technique [30] as shown in Fig 1. Kinematic data was captured with a 10-camera motion capture system (Vicon, Vantage V5 Wide Optics cameras with 22 high-powered IR LED strobes at 85nm, 240Hz). Data from the Vicon system was collected using Nexus software and then processed and analyzed using Visual 3D. Kinematic data were filtered, as suggested by Visual 3D, using a 6 Hz butterworth filter [31]. The lower kinematic model was assigned three degrees of freedom for the hip, knee, and ankle by restraining translational movement, as done by Charlton et al. [32]. The International Society of Biomechanics recommendations for coordinate systems were applied to each segment [33].

### Repetitive jumping task

The subjects performed a repeated jumping task, where they jumped ten times in a row at a self-selected jumping frequency to avoid influencing subject landing mechanics. This task was repeated three times for a total of 30 jumps. The subjects were told to jump to maximal height with their hands on their hips (Fig 1), but they were otherwise given minimal directions to perform the task.

### Variables

The flexion/extension, abduction/adduction, and internal/external rotation of the hip, knee, and ankle were chosen as the variables of interest for this study. These variables were evaluated at maximum knee flexion and processed using the entire trajectory through LDS analysis.

Grood and Suntay’s method of calculating joint angles was used to calculate knee flexion and knee abduction of both right and left legs [34].

### Lyapunov analysis

**Fig 2:**
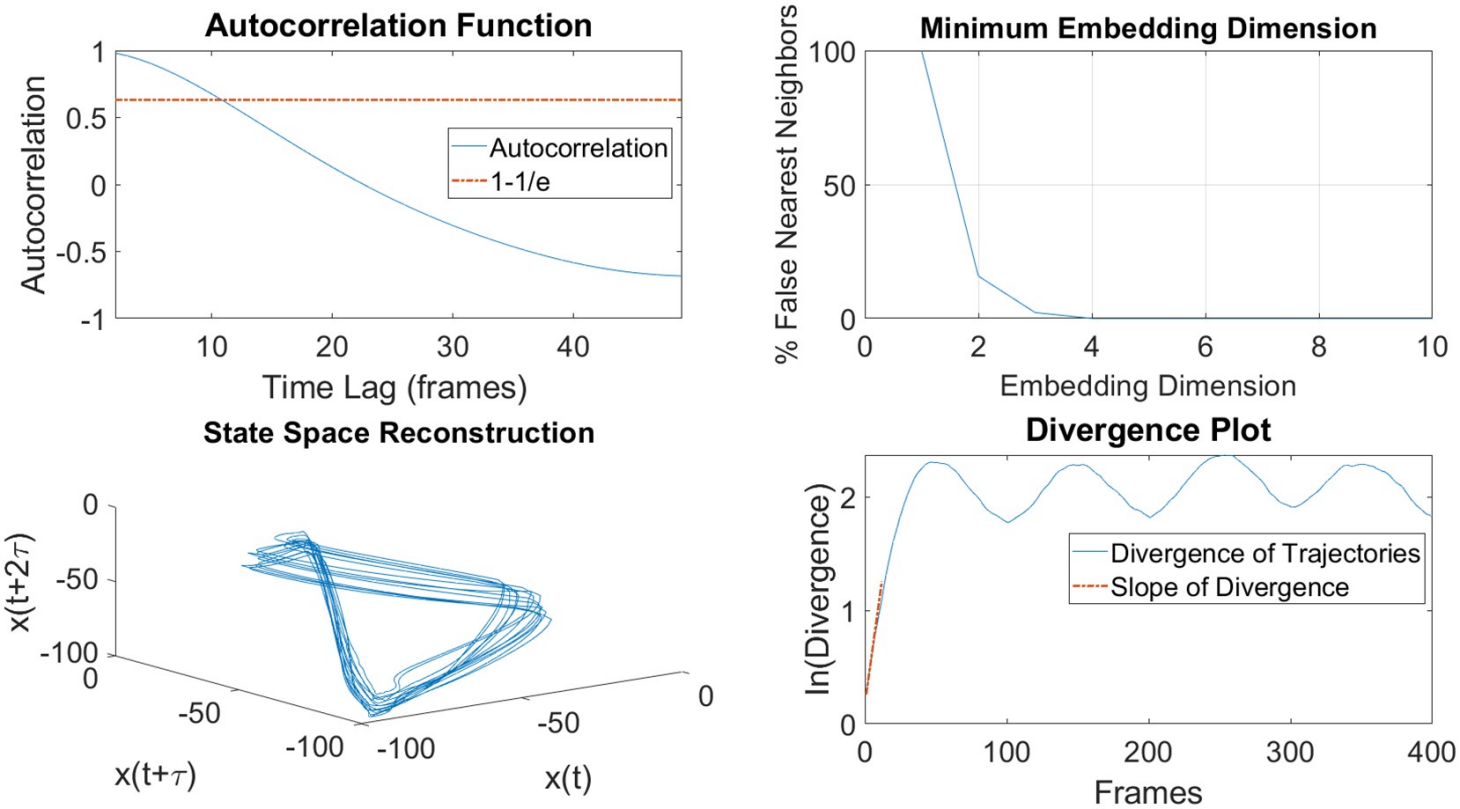
LDS calculation steps. This figure shows the steps of calculation for the LDS. Top-Left) First, a time delay is calculated using the crossing point between the autocorrelation function and 1–1/e. Top-Right) Next, the embedding dimension is calculated by finding when the percentage of false nearest neighbors goes to zero. Bottom-Left) The signal is reconstructed with the given time delay and embedding dimensions. Bottom-Right) Finally, the initial slope of the natural log of the divergence is calculated and used as the LDS.

The analysis in this paper follows Rosenstein’s method for calculating the MLE [35]. The embedding dimensions used in this study were calculated using the false nearest neighbors method, and the time delay was calculated using the autocorrelation function [36]. Embedding dimensions were calculated individually for each trial and subject, and found to be 5.12±1.85 dimensions. Time delay was similarly calculated, with the delay calculated to be 12.17±4.08 frames for all subjects. Although decimal values are presented in this set of average and standard deviation data, only integer values were used for individual embedding dimension and time delay calculations. LDS was calculated using the slope of the initial divergence data. Steps for the MLE calculation are depicted in Fig 2.

### Data processing

Jump trials were recorded for 3 sets of 10 jumps. Kinematic variables were taken at maximum knee flexion and averaged over all 30 jumps to present one value for each subject. LDS data was calculated for each set of 10 continuous jumps (3 total LDS values per subject), and the resulting 3 LDS values were averaged to result in one LDS value per subject. This method of calculation follows convention for working with similar data sets as shown by Sloot et al. [37]. To achieve signals of consistent length for LDS data analysis, kinematic data was normalized from toe-on to toe-on for each individual jump. This method of normalization is considered the most effective for data processing using Rosenstein’s method [38].

### Statistical analysis

Statistical analysis was performed using SAS software version 9.4 (SAS Institute Inc., Cary, NC, USA). A bivariate correlation was performed between variables calculated at maximum knee flexion and their corresponding LDS calculations. Additionally, two multivariate logistic regressions were run for post-hoc sorting based on previous injury status with one using only LDS variables and the other only using kinematic variables recorded at maximum knee flexion. For logistic regression analysis, previously injured subjects were given a value of “1” and previously uninjured groups were given a value of “0”. The multivariate logistic regression had stepwise entry conditions of P < 0.2 and removal conditions of P > 0.1. Finally, a set of two-sided t-tests were performed between injured and uninjured subjects for kinematic and LDS variables to explore potential differences between groups.

## Results

Table 1 shows the results from the bivariate correlation between the kinematic variables at maximum knee flexion and the same kinematic variables processed using the LDS. These results show a significant but weak correlation between ankle internal/external rotation measured at MKF and LDS.

**Table 1:** Bivariate correlation between LDS and MKF.

	r	P
**Hip Flexion/Extension**	**0.297**	**0.040**
Hip Ab/Adduction	-0.031	0.825
Hip Internal/External Rotation	0.178	0.198
Knee Flexion/Extension	-0.024	0.864
Knee Ab/Adduction	-0.213	0.121
Knee Internal/External Rotation	0.100	0.472
Ankle Flexion/Extension	-0.120	0.462
Ankle Ab/Adduction	-0.120	0.433
**Ankle Internal/External Rotation**	**0.324**	**0.018**

Correlation between LDS and corresponding kinematic variables evaluated at MKF for previously injured and uninjured subjects. Bold value indicate significance at the P < 0.05 level.

**Fig 3:**
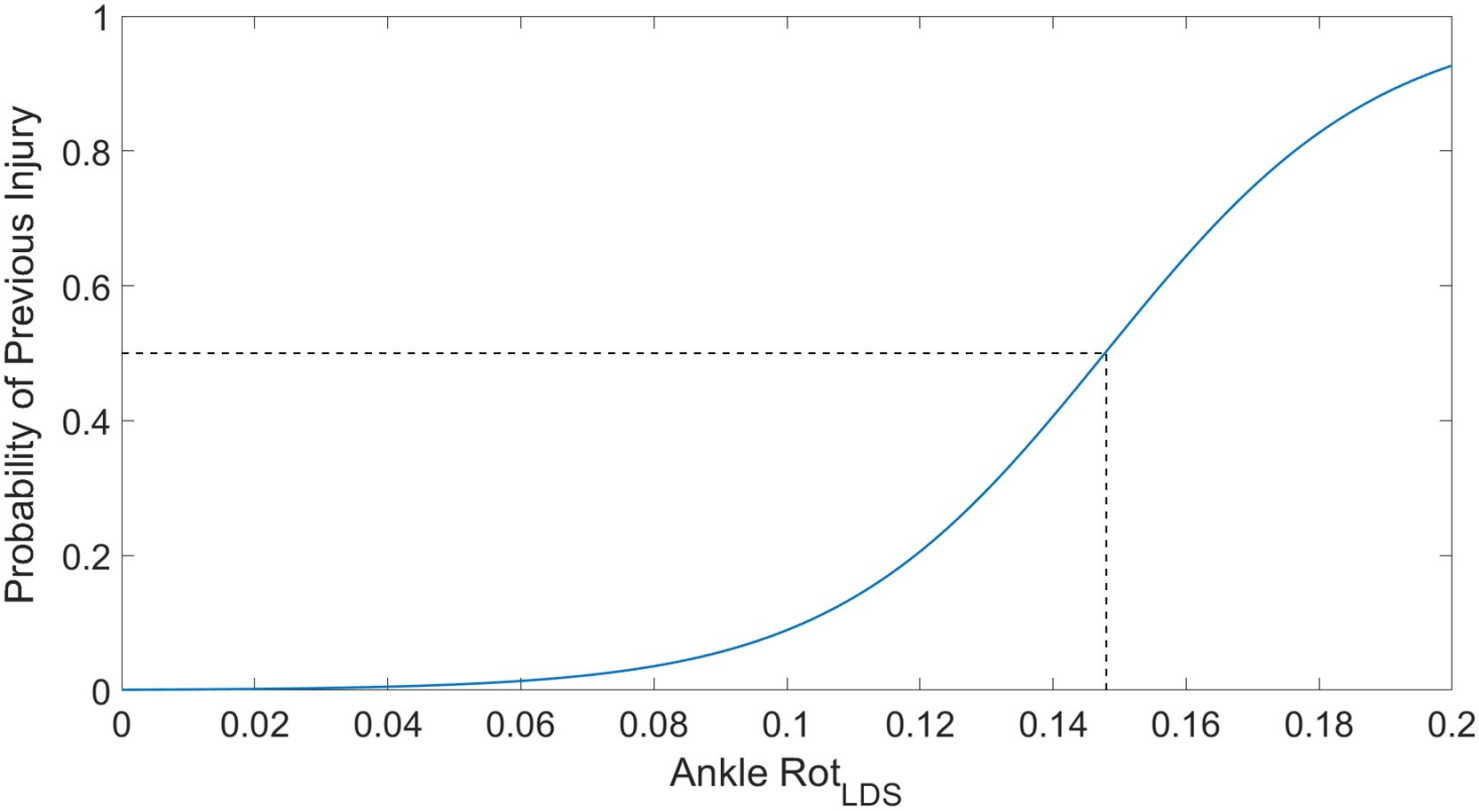
Logistic regression as a function of ankle internal/external rotation LDS. This figure shows the likelihood of belonging to the previously injured subject group based on ankle internal/external rotation LDS. The dotted lines show the approximate values at which the probability of being classified to belonging to the previously injured population is 50%. This would represent a very basic method by which the ankle internal/external rotation LDS could be used as a screening metric, and the approximate threshold at which subjects would be considered “at risk” for belonging to a previously injured group. The ROC for this figure was calculated to be 0.8407.

Fig 3 shows the results of the multivariate logistic regression when using the LDS of lower body kinematics. The LDS variables were able to successfully perform a post-hoc categorization of injured subjects with a receiver operating characteristic (ROC) of 0.8407, with ankle rotation LDS being the primary variable in this logistic regression. While a second multivariate logistic regression was performed on the kinematic variables at maximum knee flexion, there were no variables able to meet the entry and exit conditions outlined in the statistical analysis section.

**Fig 4:**
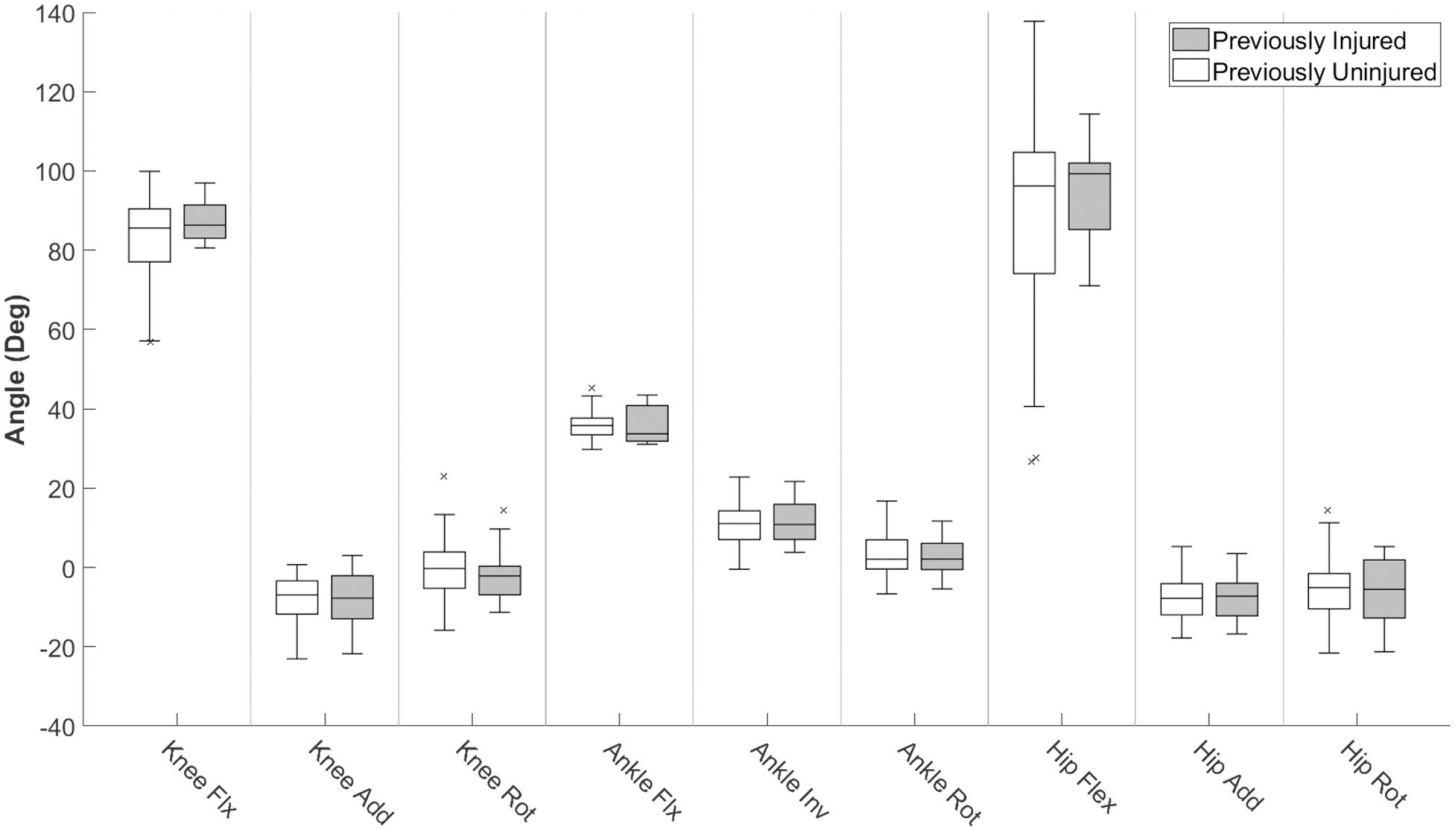
Kinematics of lower extremity at maximum knee flexion during repetitive jumps. Normative values of kinematics at MKF during a fatigue jump and their distributions. * indicates a significant difference between injured and uninjured subject groups with P < 0.05.

**Fig 5:**
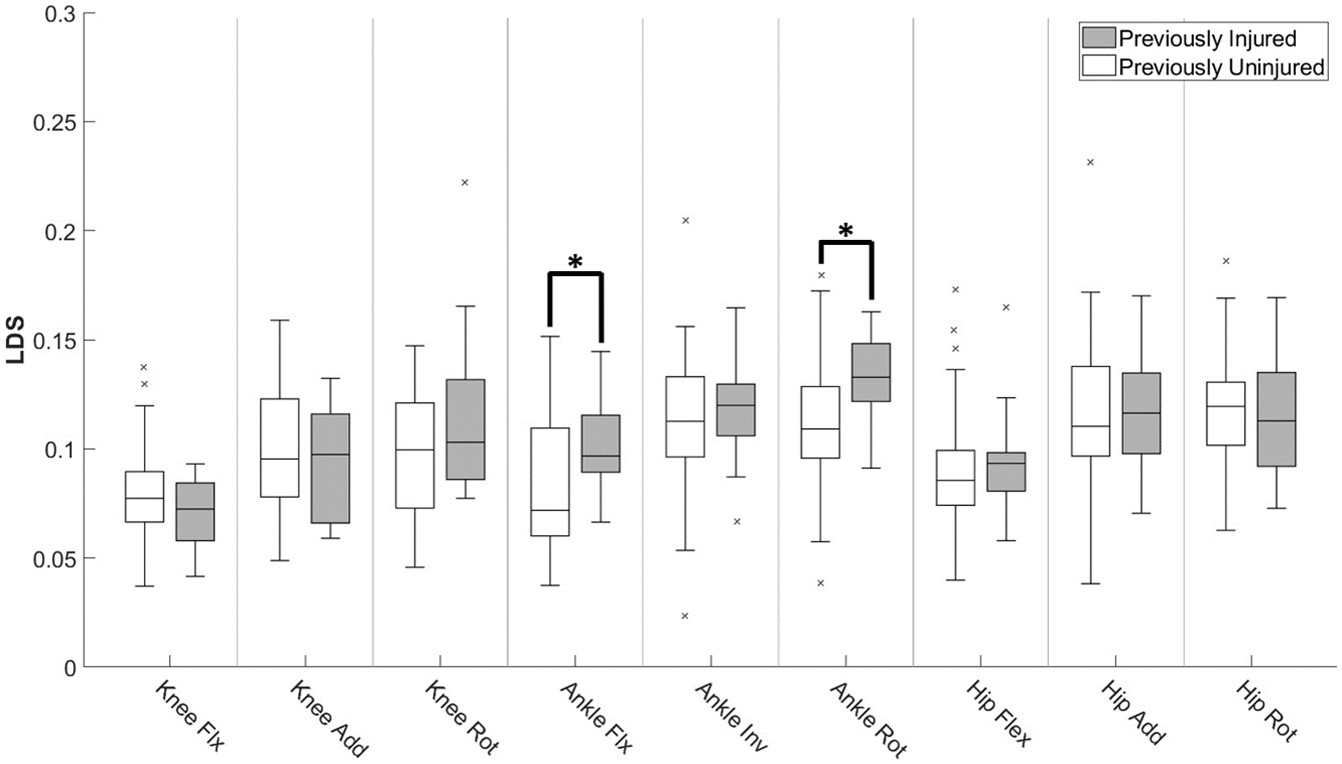
Local dynamic stability of lower extremity during repetitive jumps. Normative values of LDS during a fatigue jump and their distributions. * indicates a significant difference between injured and uninjured subject groups with P < 0.05.

Figs 4 and 5 show the differences between injured and uninjured groups for the kinematic and LDS variables respectively. There are no significant differences found for the kinematic variables, while significant differences were found for both ankle internal/external rotation and ankle flexion/extension between previously injured and uninjured groups.

## Discussion

The purpose of this study was to determine the ability of LDS variables to categorize previously injured and uninjured subjects in a post-hoc classification. The main finding was that the LDS did indeed categorize previously injured subjects at a high rate of success using a multivariate logistic regression. Conversely, the multiple traditional lower body kinematics measured at maximum knee flexion were unable to pass through the entry and exit conditions of the logistic regression.

Fig 4 shows that the lower limb kinematics at maximum knee flexion were not significantly different between previously injured and uninjured subjects, which suggests that lower limb kinematics at maximum knee flexion provides an incomplete assessment of landing strategy differences between subject groups. Furthermore, the lack of difference between groups in Table 1 shows that minimal insight to LDS can be gained through traditional kinematic analysis at maximum knee flexion during a fatigue jump. This finding shows that subjects can display similar kinematics at the bottom of a jump but still display unique movement characteristics that can be characterized with the LDS.

While the logistic regression shown in Fig 3 performed well when using LDS variables compared to the kinematic variables measured at maximum knee flexion, Fig 5 shows that there was a significant difference in ankle flexion/extension LDS between previously injured and uninjured groups as well. The increased ankle LDS for previously injured subjects indicates that a significant degree of ankle instability exists within this group, which is consistent with previous studies that identified ankle compensation as a popular movement strategy after knee injury [39–41].

Although the post-hoc design of this study does not allow for a direct comparison between other previously attempted pre-hoc injury screenings as discussed in the study by Bahr [28], it is encouraging to see that this study produced a metric with ROC = 0.84 in comparison to eccentric hamstring loading (ROC = 0.56) and medial knee displacement (ROC = 0.60). The potential for a 25–30% improvement in screening ability as well as the ability to test a wider array of non-gait movements provide a strong foundation for the authors to suggest that the LDS should be utilized in future injury screening and classification studies when possible.

### Limitations

Regarding the findings of this study, it is important to note that the data collected from the 7 previously injured subjects was gathered after the injury was sustained. Because of this, it is unclear if the findings indicate that increased LDS put subjects at a higher risk of sustaining future ACL injuries or if the decreased LDS is a result from surgery and/or rehabilitation.

Any interpretations from this dataset should be tempered when concerning populations other than women’s collegiate soccer players and a repetitive vertical jump. Because there is a limited body of work in this area, the findings presented in this paper are intended to serve as a foundation for the possibility of using the LDS as a screening metric.

### Future work

Future studies should explore the progression of LDS before, during, and after ACL injuries to understand how stability changes with respect to injury. Understanding LDS over a range of time will allow researchers to better understand the effects of stability on injury designation and whether it can be improved over time.

Because there are limited papers investigating injury prevention and LDS, there are many useful insights that could be drawn from cataloguing the LDS of various movements performed by a more general population. Although this paper focuses on athletes, there are not many studies analyzing the stability of the general population outside of traditional gait studies. For this reason, it is difficult to understand “good” and “bad” stability values outside of a direct comparison between groups—as was shown in this study.

## Conclusion

This study showed that LDS categorized previously injured subjects at a high rate of success using a multivariate logistic regression during a fatigue jump task. Although standard kinematics at maximum knee flexion were unable to identify any statistically significant differences between previously injured and uninjured subjects, the LDS was able to quantify significantly higher ankle instability for previously injured subjects. When compared with traditional screening measures, the LDS was able to correctly identify previously injured and uninjured subjects at a rate 25–30% higher than traditional screening measures. These results suggest that the LDS provides unique movement information that could allow for more effective injury screening tests, and should be critically analyzed in future studies.

## Supporting information

S1 File Lower-limb LDS datasheet. This spreadsheet shows the lower-limb LDS and kinematics and maximum knee flexion of all subjects during repetitive vertical jump task.
(XLSX)
